# Global repair is the primary nucleotide excision repair subpathway for the removal of pyrimidine-pyrimidone (6-4) damage from the Arabidopsis genome

**DOI:** 10.1038/s41598-024-53472-8

**Published:** 2024-02-08

**Authors:** Sezgi Kaya, Dugcar Ebrar Erdogan, Aziz Sancar, Ogun Adebali, Onur Oztas

**Affiliations:** 1 Department of Molecular Biology and Genetics, College of Sciences, Koc University, Istanbul, Turkey; 2 Department of Biochemistry and Biophysics, University of North Carolina School of Medicine, Chapel Hill, NC 27599 USA; 3 Molecular Biology, Genetics and Bioengineering Program, Faculty of Engineering and Natural Sciences, Sabanci University, Istanbul, Turkey

**Keywords:** Plant sciences, DNA damage and repair, Genomic instability, Genomics

## Abstract

Ultraviolet (UV) component of solar radiation impairs genome stability by inducing the formation of pyrimidine-pyrimidone (6-4) photoproducts [(6-4)PPs] in plant genomes. (6-4)PPs disrupt growth and development by interfering with transcription and DNA replication. To resist UV stress, plants employ both photoreactivation and nucleotide excision repair that excises oligonucleotide containing (6-4)PPs through two subpathways: global and transcription-coupled excision repair (TCR). Here, we analyzed the genome-wide excision repair-mediated repair of (6-4)PPs in *Arabidopsis thaliana* and found that (6-4)PPs can be repaired by TCR; however, the main subpathway to remove (6-4)PPs from the genome is global repair. Our analysis showed that open chromatin genome regions are more rapidly repaired than heterochromatin regions, and the repair level peaks at the promoter, transcription start site and transcription end site of genes. Our study revealed that the repair of (6-4)PP in plants showed a distinct genome-wide repair profile compared to the repair of other major UV-induced DNA lesion called cyclobutane pyrimidine dimers (CPDs).

## Introduction

Plants rely on solar energy for photosynthesis; however, UV component of solar radiation impairs genome stability by forming DNA lesions on plant genome in the form of dimers between adjacent pyrimidines, known as UV photoproducts. Two major types of UV photoproducts exist, namely pyrimidine-pyrimidone (6-4) photoproducts [(6-4)PPs] and cyclobutane pyrimidine dimers (CPDs). These DNA lesions hinder the activity of polymerases during transcription and DNA replication and have a detrimental effect on the growth and development of plants^1^. It is therefore vital for plants to possess effective DNA repair mechanisms to eliminate UV photoproducts, especially considering the prediction of possible rise in UV levels reaching the Earth surface^2–4^.

CPDs and (6-4)PPs, which are structurally different, represent 80% and 20% of the total DNA lesions induced by UV exposure, respectively. While CPDs contain a cyclobutane ring between the C5 and C6 positions of two adjacent pyrimidine bases, (6-4)PPs consist of a single covalent bond between C6 and C4 of two adjacent pyrimidine bases^5^. Compared to CPDs, (6-4)PPs cause a greater distortion in the structure of the DNA helix^6^. In plants, both lesions can be repaired either by photoreactivation or nucleotide excision repair^6,7^. In photoreactivation, damage-specific DNA photolyase enzymes recognize and repair CPDs and (6-4)PPs using blue-light photons as an energy source. In *Arabidopsis thaliana*, UVR2 photolyase is responsible for the repair of CPDs, whereas UVR3 photolyase repairs (6-4)PPs^8–11^.

The molecular mechanism of excision repair in plants is not yet fully understood. While plants have homologs of excision repair factors found in mammals, they lack the Xeroderma Pigmentosum Group A (XPA) protein essential for mammalian excision repair^12^, implying molecular-level differences between excision repair in plants and mammals. In mammalian cells, excision repair detects UV-induced lesions by XPA, Replication Protein A (RPA) and Xeroderma pigmentosum complementation group C (XPC) proteins. Transcription Factor IIH (TFIIH) complex is recruited to the damage site, followed by DNA strand incision with the help of Xeroderma Pigmentosum complementation group F (XPF)—Excision Repair Cross-Complementation group 1 (ERCC1) and Xeroderma Pigmentosum Group G (XPG) endonucleases. The damaged DNA strand is then replaced by polymerase and ligase activities, a process known as global repair, the subpathway of excision repair removing UV damage from all genome regions. If UV lesions occur on the transcribed strands of genes, elongating RNA polymerase II is blocked at the damage site during transcription. Cockayne syndrome group A (CSA) and CSB proteins detect stalled RNA polymerase II and recruit TFIIH complex and endonucleases, known as transcription-coupled repair (TCR). In *Arabidopsis*, transcription-coupled repair (TCR) depends on the CSA1 protein, which has been shown to interact with its homolog, CSA2 protein. However, CSA2 has a minor influence on TCR^13–15^. CSA1 is involved in CUL4-DDB1A^CSA1^ E3 ligase in plants^14^. Similarly, DDB2 interacts with the CUL4-DDB1A complex to form another E3 ligase playing a role in global repair under UV-induced stress^16^. It has been revealed that Arabidopsis plants deficient in DDB2 exhibit higher sensitivity to UV radiation compared to wild type^17^, while overexpression of Arabidopsis DDB1A enhances the plant's tolerance to UV exposure^18^.

Transcription rate across the genome mainly influences excision repair-mediated CPD repair in Arabidopsis. CPD repair level is about five times higher in the transcribed strands of genes compared to the non-transcribed strands, indicating the strong dominance of TCR in CPD repair. Additionally, the chromatin state impacts the repair of CPD lesions through excision repair, as repair is less efficient in heterochromatic regions than open chromatin regions. Furthermore, the removal of CPDs by TCR, but not global repair, shows oscillations throughout the day^19^. While the excision repair-mediated CPD repair in *Arabidopsis* has been extensively studied, there is still a lack of comprehensive knowledge regarding the genome-wide dynamics of (6-4)PP repair in plants.

The excision repair-sequencing (XR-seq) allows for the generation of a genome-wide profile of excision repair, offering precise information on the repair of specific DNA lesions at single-nucleotide resolution and at a particular timepoint^20^. By XR-seq, it becomes possible to assess the level of TCR in a specific gene by analyzing the variation in repair signals between the transcribed and non-transcribed strands of genes. XR-seq method involves immunoprecipitation of the excised DNA oligonucleotides containing the lesions (excision products) through the use of lesion-specific monoclonal antibodies. These excision products are then characterized by next-generation sequencing, and the resulting reads are aligned to the reference genome to determine the sites of excision repair activity. Here, we generated and examined the genome-wide repair map of (6-4)PPs in *Arabidopsis* seedlings by XR-seq. We found that (6-4)PPs can be repaired by TCR. However, the level of TCR during (6-4)PP repair is lower compared to CPD repair. The repair of (6-4)PP lesions exhibited distinct peaks at the promoter, transcription start site (TSS) and transcription end site (TES) of genes. Similar to CPD repair, we found that the rate of (6-4)PP repair is influenced by the chromatin state, with more efficient repair in open chromatin regions compared to regions associated with heterochromatin. Our study revealed that excision repair-mediated removal of pyrimidine-pyrimidone (6-4) damage on the Arabidopsis genome is mainly through global repair.

## Results

To understand the dynamics of excision repair-mediated (6-4)PP repair, we irradiated 10-days-old Arabidopsis seedlings with UVC (254 nm, 120 J/m^2^) and performed time course analysis of (6-4)PP-containing excision product formation at 5-, 15- and 30-min following UV irradiation. It is noteworthy that the (6-4)PP lesions on the genome were only repaired by excision repair, as we interfered with blue-light-dependent photoreactivation by keeping the seedlings under yellow light after UV irradiation. We detected generation of primary excision products, ranging from 24 to 27 nucleotides (nt) in size, at the 15-min and 30-min time points (Fig. S1A). Besides, we observed a population of short-sized excision products, approximately 18 nt in length, formed by degradation of primary excision products from the 5' end^19^. A previous study detected (6-4)PP repair activity in T87 Arabidopsis cell suspension culture 30 min after UV exposure^21^, which differs from our findings in seedlings. This difference may be caused by distinct excision repair kinetics of cell types in *Arabidopsis*^22^.

**Figure 1:**
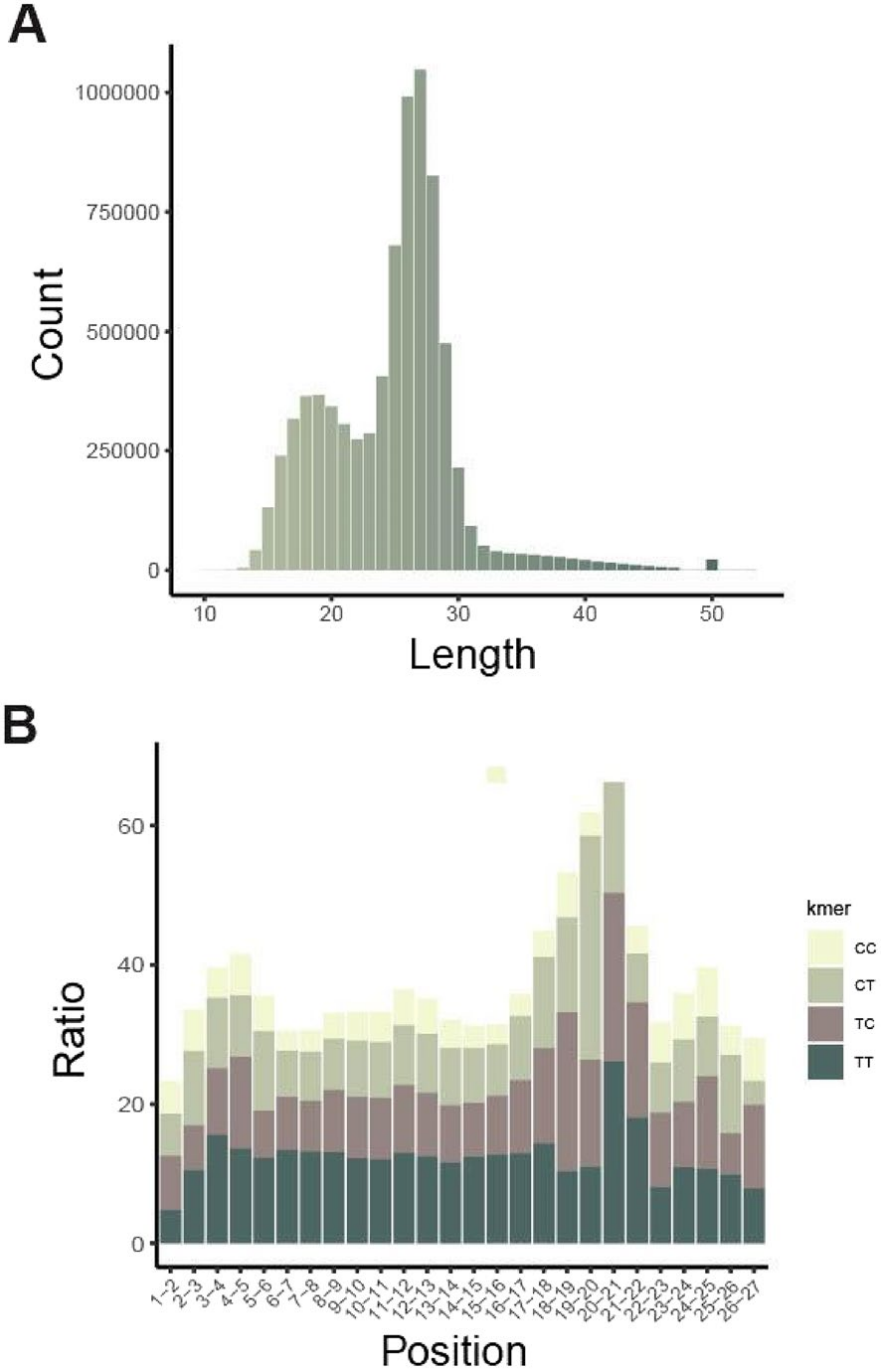
Length distribution and dinucleotide content of XR-seq reads. (**A**) Length distribution of XR-seq reads. (**B**) Distribution of CC, CT, TC and TT dinucleotides along the 27-nucleotide-long XR-seq reads*.*

To create genome-wide (6-4)PP excision repair map of *Arabidopsis* seedlings, we performed XR-seq 15 min after UV irradiation. Briefly, we immunoprecipitated the 6-4PP-containing excision products, linked adapters to them by ligation and amplified them by PCR to generate the library (Fig. S1B). We further sequenced the libraries to obtain the reads representing excision products and aligned these reads to the Arabidopsis thaliana genome^23^ to map the sites of excision repair-mediated (6-4)PP repair across the genome. Our results exhibited that two different populations of excision products in terms of length were generated during 6-4PP repair, consistent with the results of in vivo excision assay. The primary excision products were 25–28 nucleotides in length, with a peak at 27 and a population of shorter-sized excision products (16–21 nt) were present (Fig. 1A). Our analysis demonstrated that the length distribution of (6-4)PP-containing excision products are correlated with the excision products with CPD lesions^19^. Moreover, the nucleotide frequency distribution of 27-nt-long excision products revealed that the positions of 7–8 nt from the 3’ ends are pyrimidine-rich, consistent with other eukaryotes (Fig. 1B).

The excision products with (6-4)PP showed a higher frequency of cytosines at the damage site compared to CPD-containing excision products. TT and TC dipyrimidines dominated the 20th and 21st positions on the XR-seq reads with 26.1% and 24.2% abundance, respectively while the third most prevalent dipyrimidine was CT with 15.9% abundance. On the other hand, TT was by far the most dominant dipyrimidine with 42.7% abundance in the same positions of CPD XR-seq data (ZT20) reads whereas CT and TC were only 4.7% and 11.5% abundant, respectively. While UV irradiation induces the formation of CPD photoproducts mainly at the TT sites^19^, 6-4PPs are formed at a higher frequency at CC, CT and TC sites in addition to TT sites.

**Figure 2:**
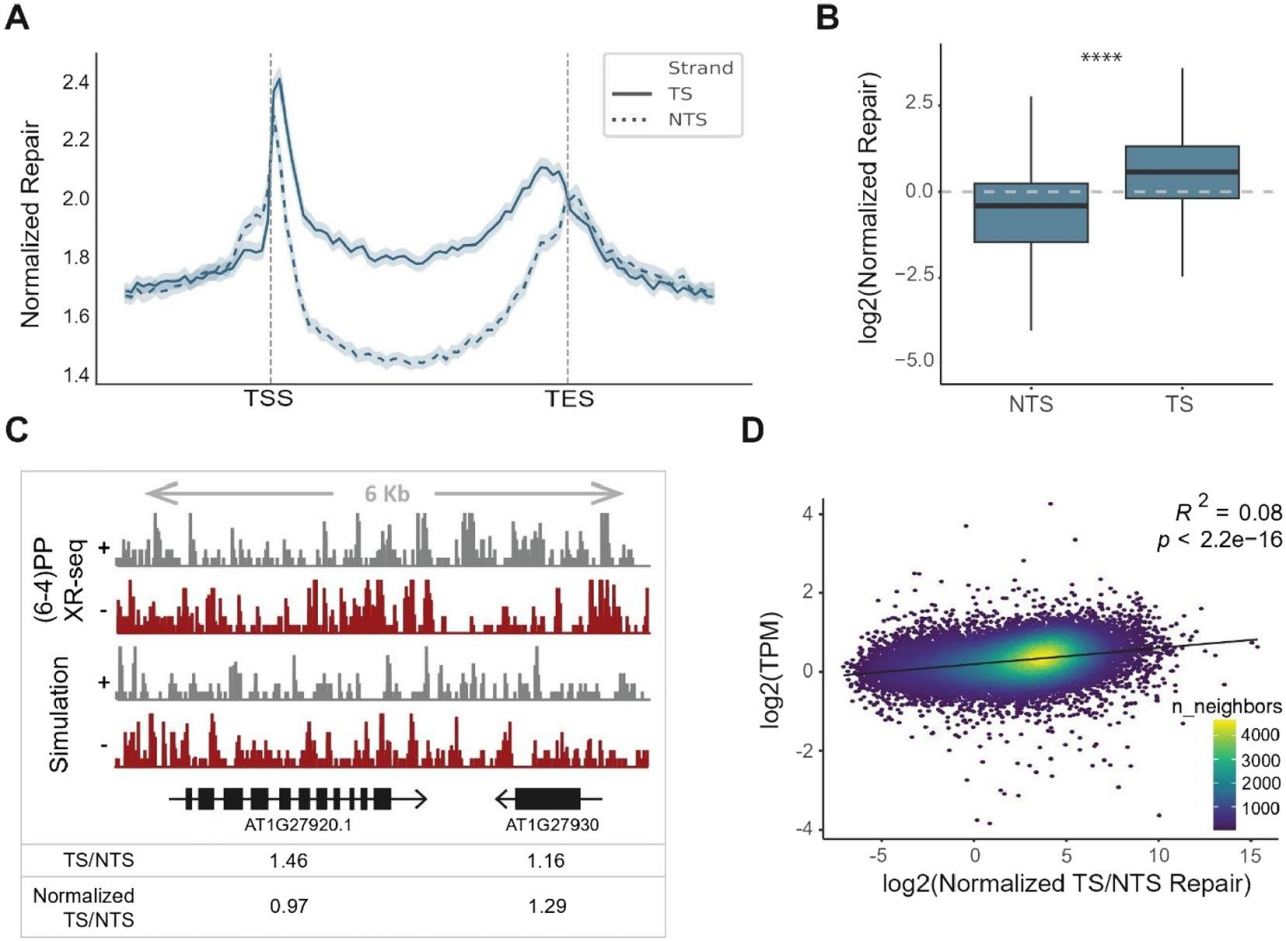
Repair profiles on Arabidopsis genome. (**A**) Normalized TS and NTS repair profiles on Arabidopsis genes and their flanking regions. Normalization of TS and NTS repair was performed by dividing the XR-seq read abundance on TS and NTS by the simulated XR-seq data abundance separately on the same genes and strands, after RPKM normalization on each genomic window. Upstream and downstream flanking regions were determined as the half of the length of its representative gene. (**B**) Normalized TS and NTS repair levels on Arabidopsis genes. Normalized repair was calculated as in (**A**) on each gene and shown in log2 scale where 0.0 represented the expected normalized repair levels due to the sequence content of the genes. (**C**) IGV screenshot of XR-seq and simulated XR-seq read distribution on two genes. TS/NTS and normalized TS/NTS ratios were shown in the below section. Normalization of XR-seq data abundance was performed as in (**B)** to obtain normalized TS/NTS repair ratios. (**D**) Correlation between normalized TS/NTS repair with the expression of the genes. TPM normalization was performed on RNA-seq data. Normalized TS/NTS ratios were obtained as in (**B**) and (**C**) and shown in log2 scale. *P*-value and correlation coefficient were calculated using Pearson correlation. Colors represent the neighboring data points.

To investigate whether *Arabidopsis* plants employ transcription-coupled repair (TCR) to remove (6-4)PP lesions, we compared repair levels in the transcribed strand (TS) and non-transcribed strand (NTS) of annotated genes. In addition, we included simulated XR-seq data which contained synthetic reads selected randomly from the genome considering the total count and sequence content of the real XR-seq data reads^24^. By normalizing the repair with the simulated repair, we obtained the normalized repair rates and eliminated the sequence content bias that might affect the repair profiles. Analyzing TS and NTS repair differences across all annotated genes revealed a slightly higher repair level in TS compared to NTS, indicating the involvement of TCR in (6-4)PP repair (Fig. 2A, B). Our results showed that repair levels at TS and NTS exhibited a peak at the transcription start site (TSS). We also detected another peak in the promoter region of genes in both TS and NTS. At the transcription end site (TES), peaks were identified in both TS and NTS, albeit with distinct positions. However, the level of TCR activity during (6-4)PP repair was lower than during CPD repair that was demonstrated in our previous study^19^. As an example, we calculated the TS/NTS ratios and determined that TS was repaired more efficiently than NTS in genes such as AT2G23430 (ICK1) and AT2G23420 (NAPRT2), as depicted in the genomic view (Fig. 2C). Next, we checked the correlation between the transcription levels and repair rates. To do that, we used RNA-seq data to calculate the expression levels (TPMs) of the genes ^25^ and compared them with the TS/NTS repair rates of each gene. The results revealed a correlation between TCR level and transcription rate during (6-4)PP repair (Fig. 2D). However, this correlation was not as pronounced as observed in the CPD repair^19^.

**Figure 3:**
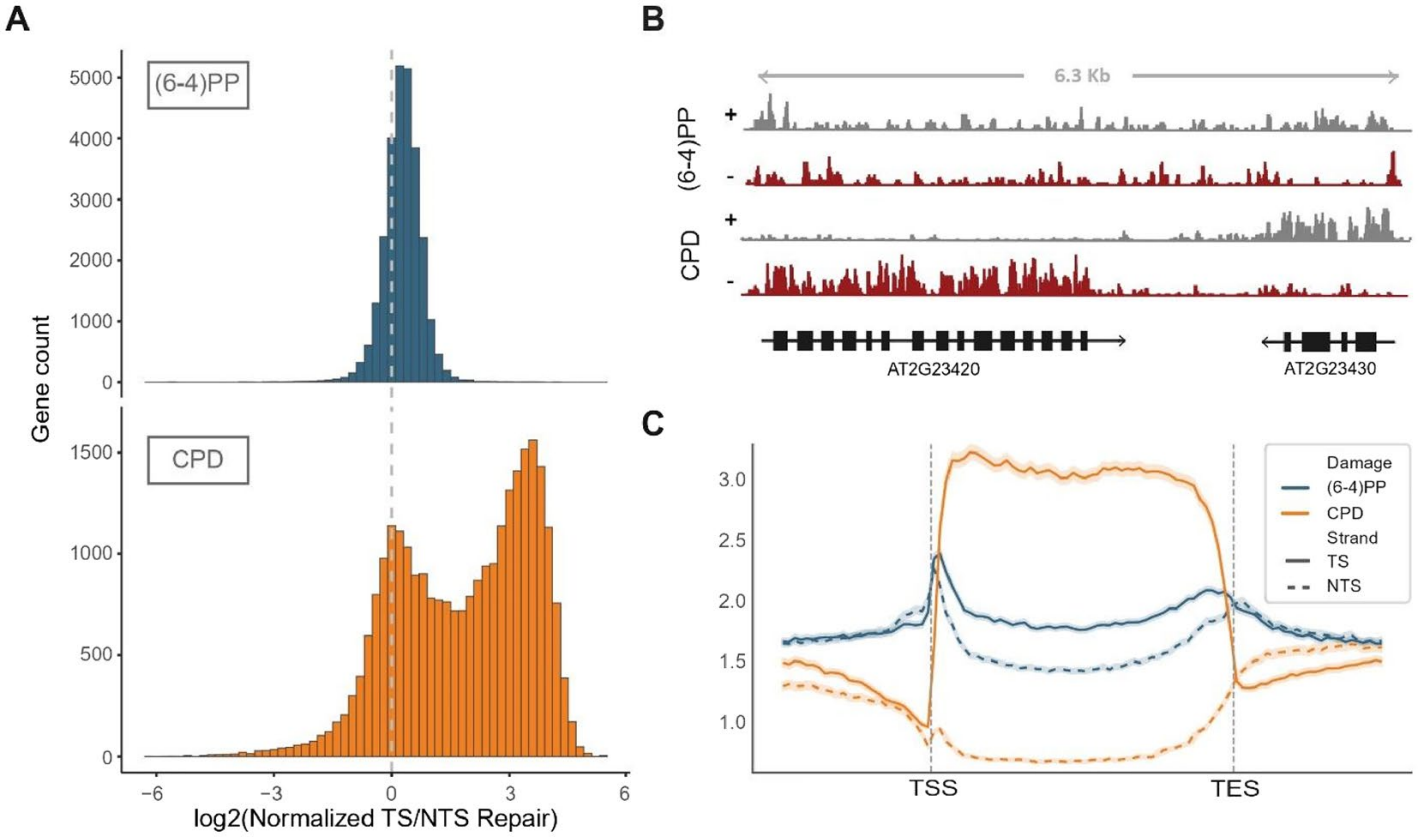
Comparison of (6-4)PP and CPD damage repair profiles on Arabidopsis genome. (**A**) Distribution of normalized TS/NTS repair ratios on Arabidopsis genes. Normalization of TS and NTS repair was performed by dividing the XR-seq read abundance on TS and NTS by the simulated XR-seq data abundance separately on each gene and strand, after RPKM normalization. (**B**) IGV screenshot of XR-seq read distribution for (6-4)PP and CPD damage types on two genes. (**C**) Normalized TS and NTS repair profiles on Arabidopsis genes and their flanking regions for (6-4)PP and CPD damage types. Normalization of TS and NTS repair was performed by dividing the XR-seq read abundance on TS and NTS by the simulated XR-seq data abundance separately on the same genes and strands, after RPKM normalization on each genomic window. Upstream and downstream flanking regions were determined as the half of the length of its representative gene.

The difference between the repair levels on TS and NTS provides insights about whether the repair at that time-point was dominated by TCR. Therefore, we compared the distribution of TS/NTS repair ratios on genes between (6-4)PP and CPD data (Fig. 3A). The repair of CPD damage has higher TS/NTS repair rates on most of the genes while TS/NTS repair ratios of (6-4)PP damage was slightly higher than 1 on most of the genes. This indicated the lower activity of TCR in (6-4)PP repair at 15-min while it is more active in CPD repair at 30-min. The screenshots of the XR-seq read distributions on two genes supported the previous observation (Fig. 3B). The (6-4)PP repair showed only a slight difference between TS and NTS while CPD repair was higher on the TS of both genes. The comparison of (6-4)PP and CPD repair profiles on Arabidopsis genes also revealed differences between the repair of these two damage types (Fig. 3C). On the genes, the repair rates of both damage types showed strand difference. However, this strand difference was much more evident in CPD repair than in (6-4)PP repair, indicating that the TCR activity was lower in (6-4)PP repair at 15-min while it is more active in CPD repair at 30-min.

**Figure 4:**
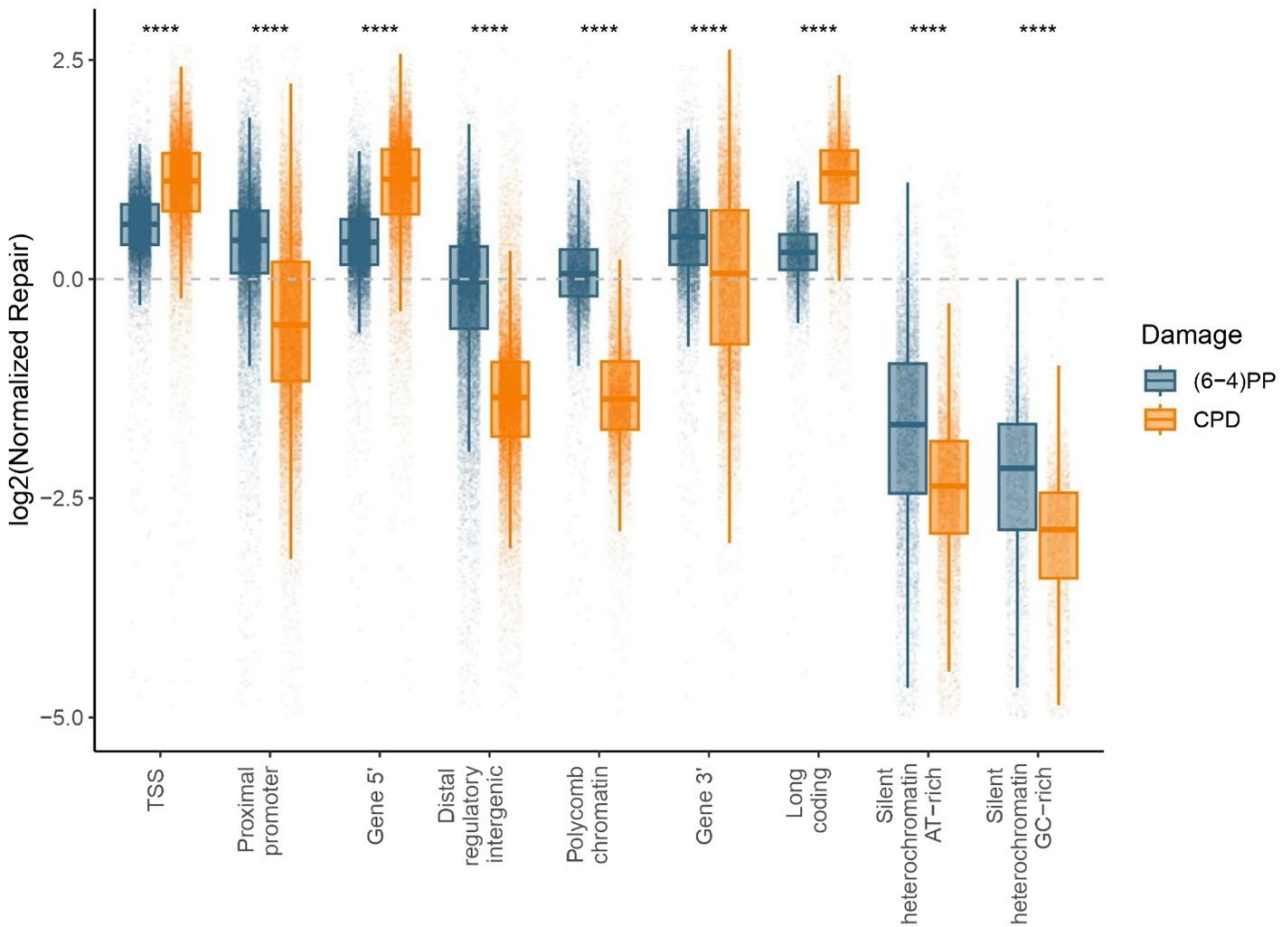
(6-4)PP and CPD repair profiles on different chromatin states on Arabidopsis genome. Normalized repair levels were calculated by dividing XR-seq read abundance on each region by the simulated XR-seq read abundance, after RPKM normalization. *P*-values were calculated using t-test (ns: *p* > 0.05; *p <  = 0.05; **p <  = 0.01; ***p <  = 0.001; ****p <  = 0.0001). ZT20 stage XR-seq data was used for CPD analysis.

Furthermore, we examined repair levels across different genomic elements identified in *Arabidopsis* genome^26^. We observed that AT-rich and GC-rich heterochromatin regions of the genome exhibited slower repair compared to distal regulatory intergenic genomic regions with euchromatin signatures (Fig. 4), indicating that the epigenetic state influences the rate of (6-4)PP repair. We found similar (6-4)PP repair levels in transcribed regions, such as the 5' and 3' ends of genes, unlike CPD repair, indicating that the repair level difference between the 5' and 3' ends of genes in CPD repair is due to strong TCR. In (6-4)PP repair, where TCR is less prominent, this difference diminishes. In conclusion, for (6-4)PP repair, global repair mechanisms play a more significant role than TCR. The main factor determining (6-4)PP repair is the epigenetic state, while transcription rate plays a more significant role in CPD repair^19^.

## Discussion

As sessile organisms, plants are exposed to the UV component of sunlight during photosynthesis. While efficiently capturing blue and red light by chlorophylls, they must protect their genome from UV radiation. Thanks to the stratospheric ozone layer, only UV-A (320–400 nm) and UV-B (280–320 nm) reach the Earth's surface, UV-C (100–280 nm), the most damaging type, is blocked. UV-B causes the formation of UV photoproducts on plant’s genome^9^. Plants possess flavonoids and carotenoids to block UV penetration into deep tissues and eliminate the oxidative stress caused by UV exposure^27–29^. In order to maintain their genome integrity, plants employ two DNA repair mechanisms—photoreactivation, active only during the day due to its dependence on blue light, and nucleotide excision repair, functional all day. This all-day functionality of excision repair presumably becomes important for removing UV damage occurring at dusk. Since photolyases rely on blue light for their functionality, therefore at dusk there is a likelihood that photolyases may be incapable of eliminating UV damage accumulated prior to this period. As a result, excision repair becomes essential at such timepoints to ensure the maintenance of genome stability.

Our previous analysis on CPD excision repair demonstrated a high level of TCR, meaning the excision repair is preferentially more active at transcribed strands of genes. The correlation between transcription rate and TCR shows a higher repair rate in the genes with higher transcription level. By this way, under UV stress excision repair factors are, presumably, efficiently directed to the UV damages on the genes required to maintain the biological processes vital at that time. There is no evidence yet suggesting a preference for a specific genomic region in photoreactivation. Moreover, the removal of UV damage in heterochromatic regions requires chromatin relaxation enabling access to the damage site. While this regulation has been observed in excision repair, although it requires a more detailed investigation, there is no evidence, and it is likely impossible, that photoreactivation, as a single-protein DNA repair mechanism, initiates chromatin remodeling mechanisms independently to access the damaged sites. Therefore, understanding the molecular mechanism and dynamics of plant excision repair is crucial.

Our studies showed distinct genome-wide repair patterns for (6-4)PP and CPD lesions in *Arabidopsis*. The key difference is in the prevalence of TCR during excision repair-mediated CPD repair, whereas global repair is the primary pathway for eliminating 6-4PPs. These findings show that the activity of excision repair in plants is specific to the damage type. Excision repair's significance for genome integrity probably extends beyond UV damage, since *Arabidopsis* lacking functional excision repair mechanisms accumulating mutations even without UV exposure^9^. This emphasizes the critical role of excision repair in eliminating bulky DNA adducts caused by DNA-damaging factors other than UV, necessitating further screening for these factors.

During CPD repair, TCR plays a crucial role in sustaining gene transcription under UV stress. Interestingly, our analysis of (6-4)PP excision repair revealed a lower presence of TCR. However, we found that the repair level for (6-4)PP peaks at promoter sites, TSS, and TES regions, a pattern absent in CPD repair. This suggests that the repair bias during (6-4)PP repair is enabled by targeting excision repair to the sites for transcription initiation and termination of specific genes important for plant fitness under UV stress.

UV radiation is among the important abiotic factors impacting plant life. It is known that the terrestrialization of plants became possible with the evolution of UV resistance mechanisms^30,31^. In addition to directly causing damage on the molecules of the cell, UV stress imposed on plants probably results in unnecessary energy loss, diverting resources that could otherwise contribute to growth and development. UV stress level depends on the trajectory of ozone layer, which is influenced by climate change-related factors, such as increased wildfires generating aerosols, polar ice melting, the change in the species distribution across the globe and deforestation^32^. Therefore, the breeding of plants for UV resistance should be prioritized under the environmental conditions with the unpredictable nature of UV levels reaching the Earth's surface. This becomes more crucial considering the potential for mutation accumulation, threatening the genome stability of cultivated crops, and posing risks to yield, crop quality, and economic stability.

## Methods

### Plant materials and growth conditions

Ten-day-old seedlings of Arabidopsis thaliana Columbia (Col-0) accession were used in this study. Plants were grown under a long-day condition (16 h light/8 h dark) with a cool white, fluorescent light at 24 °C. Eight milligrams of seeds for each sample were surface-sterilized, and stratified for 2 days at 4 °C, and then planted on a Murashige and Skoog plate. The seedlings were collected at circadian timepoint of ZT20.

### Excision assay

Ten-day-old seedlings were irradiated with 1 J/(m2s) UVC (254 nm) for 2 min (120 J/m2 UVC). After 15-min incubation under yellow light at 24 °C, the seedlings were frozen with liquid nitrogen, and were ground using mortar and pestle. The resulting powder was resuspended in 400 µl of STES buffer (200 mM Tris⋅HCl pH 8.0, 500 mM NaCl, 0.1% SDS, 10 mM EDTA) and 400 µl of phenol:chloroform (20:1). The sample was homogenized by vortexing with acid-washed glass beads for 30 min at 4 °C, followed by centrifugation at 14,000 rpm for 10 min at room temperature. The supernatant was treated with 10 µl of RNAseA (R4642; Sigma) for 1 h at 37 °C, then with 10 µl of proteinase K (P8107S; NEB) for 1 h at 60 °C. The excision products were obtained by ethanol precipitation and purified by immunoprecipitation with a (6-4)PP-specific antibody obtained from Cosmo Bio. (NMDND002). These fragments were 3′-end radiolabeled with [α-32P]-3′-deoxyadenosine 5′-triphosphate (cordycepin 5′-triphosphate) (Perkin-Elmer) by terminal deoxynucleotidyl transferase (NEB), and visualized on an 11% sequencing gel.

### XR-seq library preparation

Excision products were purified as in excision assay. 5′ and 3′ adapters compatible with the Illumina TruSeq Small RNA protocol were ligated to excision products. Ligation products were immunoprecipitated with (6-4)PP antibodies, and photoreversed with purified (6-4)PP photolyase to remove (6-4)PPs interfering with following PCR approaches. Analytical PCR was performed using one percent of the sample to decide the minimum number of cycles required for preparative scale PCR amplification. The repaired ligation products were PCR-amplified using 50- and 63-nt-long primers adding specific barcodes compatible with the Illumina TruSeq Small RNA kit. The correct size PCR products representing the library were gel purified, and then sequenced in the Illumina HiSeq 4000 platform, and single-end 50-nt reads were generated.

### XR-seq data preprocessing

Raw data was processed using the snakemake pipeline available in Github (https://github.com/CompGenomeLab/xr-ds-seq-snakemake). Briefly, Cutadapt (v4.1)^33^ was used to trim the adaptor sequence (TGGAATTCTCGGGTGCCAAGGAACTCCAGTNNNNNNACGATCTCGTATGCCGTCTTCTGCTTG) from the reads. Trimmed reads were aligned on Arabidopsis genome Araport11 with Bowtie2 (v2.4.1)^34^. SAM files were subjected to quality filtering with samtools (v1.10) (-q 20) and converted to BAM format^35^. After duplicate elimination with Picard (v2.27), BEDTools (v2.29.0) was used to obtain BED files^36^. Simulated XR-seq data was created using Boquila algorithm^24^ that is embedded in the snakemake pipeline**.**

### Read length distribution and nucleotide frequency

The read length distributions and nucleotide abundance plots were plotted using R ggplot2 package. The 27-nucleotide reads were used for the nucleotide abundance plot.

### Screenshots

BED files were converted to BedGraph format with RPM normalization using BEDTools (v2.29.0). BedGraph files were converted to BigWig format using UCSC tools bedGraphToBigWig utility. BigWig files were then visualized using Integrative Genomics Viewer (IGV).

### Repair profiles on Arabidopsis genes

The regions including the protein-coding genes of Araport11 genome and upstream and downstream regions in the half-length of each gene were divided into 100 equal bins. Each bin was checked for intersections with the XR-seq data for CPD and (6-4)PP damage types strand-specifically, using BEDTools intersect. The replicates of both data were merged before intersecting. The same intersections were checked between the bins and simulated XR-seq data of both damage types. The intersecting reads were counted for both real and simulated data on both strands and subjected to RPKM normalization, separately. The real XR-seq data RPKMs were divided by the simulated XR-seq data RPKMs to obtain the normalized repair rates separately for both damage types. The normalized repair rates were plotted using the Phyton package Seaborn (v0.10.1).

TS/NTS repair rates were calculated by checking the intersections between the complete protein-coding gene regions and real and simulated XR-seq data on both TS and NTS. Counts were subjected to RPKM normalization and real XR-seq RPKMs were divided by simulated XR-seq RPKMs on each strand of each gene to obtain normalized repair rates separately for TS and NTS. Finally, normalized repair on TS was divided by the normalized repair on NTS, to calculate TS/NTS repair rates for each gene. The boxplots were generated using R package ggplot2^37^.

### Correlation between gene expression and repair

Three replicates of RNA-seq data of wild-type nine-day-old seedlings of Arabidopsis Col-0 plants at ZT22 stage retrieved from Rugnone et al.^25^ was aligned together on Arabidopsis Araport11 genome with STAR (v2.7.10b)^38^. Expression levels of the transcripts were calculated using Salmon (v0.13.1)^39^. The longest of the isoform transcripts was selected as the representative and the TPM expression of that isoform was assigned to the annotated gene. The correlation between TPM and TS/NTS repair of each gene was plotted in R package ggplot2^37^.

### Repair on chromatin states

Arabidopsis chromatin states were retrieved from a previous study^26^ and genomic segments of each state were intersected with the real and the simulated XR-seq data, without respect to strand. On each segment, following the RPKM normalization, XR-seq counts were divided by the simulated XR-seq counts to obtain normalized repair rates. R package ggplot2^37^ was used to plot the normalized repair.

### Supplementary Information

Supplementary Figure S1.

## Data Availability

All sequencing data that support the findings of this study have been deposited in the National Center for Biotechnology Information Gene Expression Omnibus (GEO) and are accessible through the GEO Series accession number “GSE243671”. All other relevant data are available from the corresponding authors on request. The secure token (stcnwuqipdejbkt) has been created to allow review of record GSE243671 while it remains in private status.
