# Supplementary material for: Global repair is the primary nucleotide excision repair subpathway for the removal of pyrimidine-pyrimidone (6-4) damage from the Arabidopsis genome
Source: Sci Rep. 2024 Feb 8;14:3308. doi: 10.1038/s41598-024-53472-8 (PMC10853524; doi:10.1038/s41598-024-53472-8)
Supplement: Supplementary file 1 — Supplementary Figure S1. [file 41598_2024_53472_MOESM1_ESM.pdf]

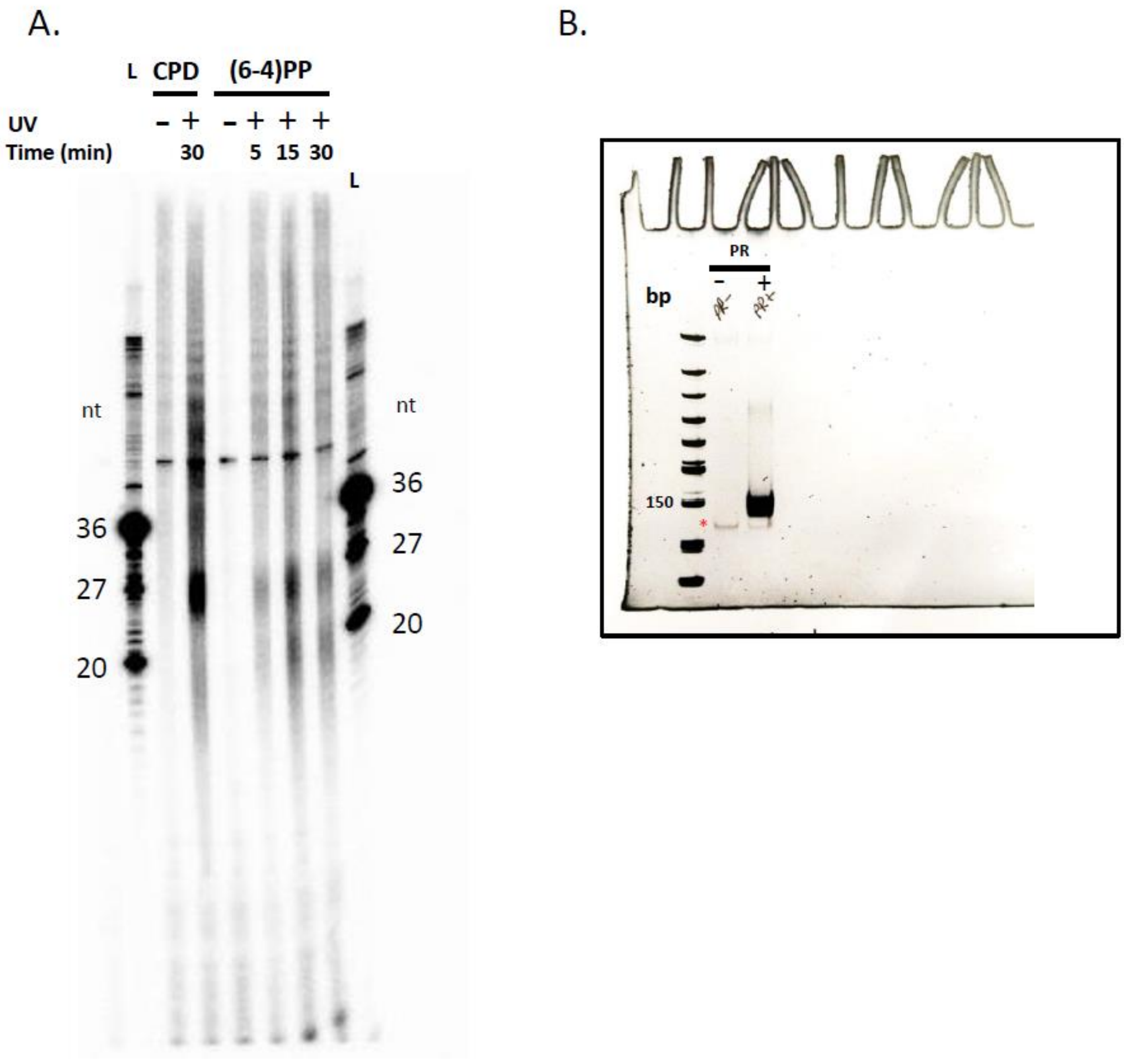

Figure-S1: Nucleotide excision repair eliminates (6-4)PPs in *Arabidopsis*. A) Detection of CPD and (6-4)PP -containing excision products in UV-irradiated seedlings by immunoprecipitation with anti-CPD-DNA and anti-(6-4)PP-DNA antibodies at different timepoints following UV irradiation. L: DNA ladder, nt: nucleotide **B)** Polyacrylamide gel showing the XR-seq dsDNA library of the excision repair products after adapter ligation, photoreactivation (PR), and PCR. The asterisk indicates a non-specific band. bp: base pairs
